# Cognitive impairment is associated with mitochondrial dysfunction in peripheral blood mononuclear cells of elderly population

**DOI:** 10.1038/s41598-020-78551-4

**Published:** 2020-12-08

**Authors:** Nattayaporn Apaijai, Sirawit Sriwichaiin, Arintaya Phrommintikul, Thidarat Jaiwongkam, Sasiwan Kerdphoo, Sirintorn Chansirikarnjana, Nisakron Thongmung, Usanee Mahantassanapong, Prin Vathesatogkit, Chagriya Kitiyakara, Piyamitr Sritara, Nipon Chattipakorn, Siriporn C. Chattipakorn

**Affiliations:** 1 Neurophysiology Unit, Cardiac Electrophysiology Research and Training Center, Faculty of Medicine, Chiang Mai University, Chiang Mai, 50200 Thailand; 2 Center of Excellence in Cardiac Electrophysiology Research, Chiang Mai University, Chiang Mai, 50200 Thailand; 3 Cardiac Electrophysiology Unit, Department of Physiology, Faculty of Medicine, Chiang Mai University, Chiang Mai, 50200 Thailand; 4 Department of Internal Medicine, Faculty of Medicine, Chiang Mai University, Chiang Mai, 50200 Thailand; 5 Department of Medicine, Faculty of Medicine, Ramathibodi Hospital, Mahidol University, Bangkok, 10400 Thailand; 6 Research Center Faculty of Medicine, Ramathibodi Hospital, Mahidol University, Bangkok, 10400 Thailand; 7 Medical and Health Division, Electricity Generating Authority of Thailand, EGAT 53 Moo 2 Charansanitwong Road, Bangkruai, 11130 Nonthaburi Thailand; 8 Department of Oral Biology and Diagnostic Sciences, Faculty of Dentistry, Chiang Mai University, Chiang Mai, 50200 Thailand

**Keywords:** Cognitive ageing, Neuronal physiology, Dementia

## Abstract

Cognitive impairment is commonly found in the elderly population. Evidence suggests that mitochondrial function in lymphocytes are potential biomarkers in the progression of neurodegeneration, as peripheral mitochondrial function is associated with mild cognitive impairment (MCI) in the elderly population. Therefore, we hypothesize that impaired mitochondrial ATP production and oxidative stress in peripheral blood mononuclear cells (PBMCs) are associated with cognitive impairment in the elderly population. Data were collected from 897 participants from the EGAT (The Electricity Generating Authority of Thailand) cohort. The participants were classified to be in the normal cognition group (n = 428) or mild cognitive impairment group (n = 469), according to their MoCA score. The association of mitochondrial function and cognitive status was analyzed by binary logistic regression analysis. MCI participants had higher age, systolic blood pressure, waist/hip ratio, and lower plasma high- and low-density lipoprotein cholesterol levels, when compared to the normal cognition group. In addition, estimated glomerular filtration rate were lower in the MCI group than those in the normal cognition group. Collectively, MCI is associated with mitochondrial dysfunction in PBMCs as indicated by decreasing mitochondrial ATP production, increasing proton leak, and oxidative stress, in the elderly population, independently of the possible confounding factors in this study.

## Introduction

The elderly population (age > 65 years old) is continuing to increase globally. It has been shown that the elderly accounted for 9% of the population in 2018, a statistic that is projected to be 17% by 2050^1^. Aging is strongly associated with cognitive impairment, which can progress to dementia^2^. The risk factors associated with cognitive impairment include increased age, and having the APOE-e4 gene, as well as other medical conditions, including metabolic disturbances and depression^3^. Due to the association between cognitive impairment and quality of life^4^, screening tools have been developed for early diagnosis of cognitive impairment including the Mini-Mental State Examination (MMSE) and the Montreal Cognitive Assessment (MoCA). A recent report showed that the MoCA test had higher sensitivity and specificity than the MMSE in the testing of cognitive function in elderly people (age > 60 years old)^5^. The MoCA test is a 30-point test for assessing short-term memory recall, visuospatial abilities, multiple aspects of executive functions, attention, concentration, working memory, language, and orientation. MoCA is a sensitive and reliable tool for the detection of mild cognitive impairment (MCI). The cutoff point for a screening of MCI is a MoCA score of < 26^6,7^. However, the limitation of MoCA on its accuracy for MCI may be compromised by several confounding factors, including education and age. Therefore, search for any potential biomarkers for MCI, particularly in elderly, should be investigated.

Mitochondrial dysfunction has been identified as a mechanism responsible for cognitive impairment^8,9^. Previous studies in animal models of MCI demonstrated increased brain mitochondrial oxidative stress level^10^, reduced mitochondrial integrity^11^, impaired mitochondrial electron transport systems^12^, and increased cellular energy depletion. Peripheral blood mononuclear cells (PBMCs) are being used to investigate the involvement of mitochondria in cognitive function in patients with MCI^13^. The results from these investigations demonstrated that MCI patients had a lower level of mitochondrial deoxyribonucleic acid (DNA) when compared with healthy subjects^13^. In addition, mitochondria are the main source of cellular energy production, the energy being released in the form of adenosine triphosphate (ATP) through the five complexes of the electron transport chain^14^. Oxygen is used to support ATP synthesis, and mitochondrial-linked ATP production can be represented as an oxygen consumption rate^15^. During ATP production, reactive oxygen species (ROS) are produced from the electron transport chain, mostly from complex I and III^16^. ROS are essential for maintaining metabolic processes, mediating cell growth, and immunity^17^. It is also known that an excessive ROS level can induce mitochondrial dysfunction, and DNA damage, resulting in cell death^18^. However, the association between mitochondrial respiration, mitochondrial-linked ATP production, and mitochondrial oxidative stress obtained from the PBMCs in elderly subjects, with or without MCI, has never been investigated. The present study hypothesizes that mitochondrial-linked ATP production and mitochondrial oxidative stress from the PBMCs can be potential biomarkers representing a cognitive impairment in an elderly population.

## Results

### Participants and demographic data

1199 participants from the Electricity Generating Authority of Thailand (EGAT) were enrolled in this study, we excluded 302 participants who had underlying diseases, dementia (which defined by Thai MMSE score ≤ 14 for illiterates, ≤ 17 for elementary school, or ≤ 22 for over elementary school), or missing MMSE or MoCA score data. The remaining 897 participants were divided into two groups, as normal cognition and MCI groups. Participants who had a MoCA score ≥ 26 were added to the normal cognition group, while participants who have MoCA score < 26 were categorized into the MCI group. The activities of daily living (ADL) and instrumental activities of daily living (IADL) were slightly lower in the MCI group than normal cognition group (p < 0.001 and p < 0.001, respectively). However, these differences did not show any clinical significance, as indicated by the normal activities of daily living in both groups. Demographic data showed that participants in the MCI group were older than in the normal cognition group (p < 0.001). Data on systolic and diastolic blood pressure (SBP and DBP), Body Mass Index (BMI), and Waist to Hip Ratio (WHR) showed that participants in the MCI group had higher SBP and WHR than the normal cognition group (p < 0.05, p < 0.01, respectively); however, DBP and BMI were not different between groups. For underlying diseases, participants with DM and dyslipidemia were comparable between the MCI and normal cognition groups, but there were more participants with hypertension in the MCI group when compared with normal cognition group (62% vs. 55%, p < 0.05) (Table 1).

**Table 1:** Demographic data of participants.

Variables	Normal (n = 428)	MCI (n = 469)	p-value
Age (years)	72 (4)	74 (4)	**< 0.001**
Sex (Male)	139 (32.5%)	120 (25.5%)	**0.022**
Systolic blood pressure (mmHg)	145 (18)	148 (19)	**0.003**
Diastolic blood pressure (mmHg)	75 (10)	74 (10)	0.725
Body mass index (kg/m^2^)	24.28 (3.47)	24.33 (4.01)	0.860
Waist to hip ratio	0.93 (0.07)	0.94 (0.06)	**0.001**
MoCA score	27.83 (1.33)	21.60 (3.12)	**< 0.001**
ADL score	19.85 (0.48)	19.67 (0.95)	**< 0.001**
IADL score	7.97 (0.16)	7.83 (0.16)	**< 0.001**
**Education**			
Primary school (n, %)	13 (3.0%)	60 (12.8%)	**< 0.001**
High school (n, %)	27 (6.3%)	84 (17.9%)	
Vocational (n, %)	109 (25.5%)	154 (32.8%)	
Bachelor (n, %)	225 (52.6%)	152 (32.3%)	
Master and above (n, %)	54 (12.6%)	20 (4.3%)	
**Underlying diseases**			
Diabetes mellitus (n, %)	89 (20.9%)	115 (25.1%)	0.151
Hypertension (n, %)	234 (55.1%)	283 (61.8%)	**0.047**
Dyslipidemia (n, %)	258 (60.8%)	268 (58.6%)	0.536
**Blood parameters**			
White blood cell (cell/mm^3^)	6211 (1581)	6379 (1680)	0.123
Hemoglobin (g/dL)	13.75 (1.40)	13.65 (1.32)	0.254
Percent Neutrophil (%)	57 (9)	58 (9)	0.368
Percent lymphocyte (%)	32 (8)	32 (8)	0.261
Triglyceride (mg/dL)	117 (52)	118 (60)	0.703
HDL-C (mg/dL)	61 (16)	58 (17)	**0.043**
LDL-C (mg/dL)	127 (39)	120 (35)	**0.005**
VLDL-C (mg/dL)	23 (10)	24 (12)	0.683
AST (U/L)	25 (9)	25 (16)	0.929
ALT (U/L)	23 (12)	23 (26)	0.788
ALP (U/L)	68 (21)	69 (24)	0.652
Glucose (mg/dL)	100 (22)	104 (27)	**0.019**
Albumin (g/dL)	4.70 (0.29)	4.65 (0.30)	**0.004**
Globulin (g/dL)	2.76 (0.45)	2.86 (0.43)	**0.001**
Albumin/Globulin ratio	1.76 (0.38)	1.67 (0.32)	**< 0.001**
eGFR (mL/min/1.73m^2^)	62.58 (20.48)	57.05 (19.87)	**< 0.001**

Continuous variables are presented as mean (standard deviation) and are compared between normal and MCI participants by Student’s t-test. Categorical variable is presented as number with percent and are compared by the chi-square test. Bold values represent a statistical significance.

*ADL* activities of daily living, *ALP* alkaline phosphatase, *ALT* alanine aminotransferase, *AST* aspartate aminotransferase, *eGFR* estimated glomerular filtration rate, *HDL-C* High-density lipoprotein cholesterol, *IADL* instrumental activities of daily living, *LDL-C* Low-density lipoprotein cholesterol, *MoCA* Montreal cognitive assessment, *VLDL-C* Very low-density lipoprotein cholesterol.

Fasting plasma triglyceride, high-density lipoprotein (HDL-C), low-density lipoprotein (LDL-C), very low-density lipoprotein (VLDL-C), and glucose were determined. Our results showed that plasma glucose levels were increased in participants with MCI (p < 0.05) when compared with the normal cognition group. Although we found a significant reduction in HDL-C (p < 0.05) in MCI subjects, LDL-C was also reduced (p < 0.01). Triglyceride and VLDL-C levels were not different between groups (Table 1).

The blood was also used to measure kidney function markers, and data showed that in MCI participants, estimated glomerular filtration rate (eGFR) was decreased when compared with normal cognition participants, suggesting that our MCI subjects had a decrease in kidney function (Table 1). The complete blood count and liver function parameters were assessed. Albumin/globulin ratio was decreased in MCI participants (p < 0.05). Parameters from complete blood count and other liver function parameters were comparable between groups.

### The mitochondrial function between normal cognition participants and MCI participants

Data from mitochondrial respiration parameters and oxidative stress parameters are shown in Table 2. For mitochondrial function parameters, only the oxygen consumption rate (OCR) value of mitochondrial-linked ATP production was lowered in the MCI group compared to the normal cognition group (p < 0.05), while the OCR values of other parameters including non-mitochondrial respiration, mitochondrial basal respiration, proton leak, maximal respiration, and spare respiratory capacity were comparable between MCI and normal cognition groups.

**Table 2:** Comparison of mitochondrial respiration parameters and mitochondrial oxidative stress parameters between normal and MCI participants.

Variables	Normal (n = 428)	MCI (n = 469)	p-value
**Mitochondrial respiration parameters**			
Non-Mitochondrial respiration (OCR, pmol/min)	24.00 (10.33)	23.86 (10.11)	0.857
Mitochondrial Basal respiration (OCR, pmol/min)	57.73 (33.17)	56.00 (33.34)	0.476
Mitochondrial proton leak (OCR, pmol/min)	12.26 (10.78)	12.99 (12.24)	0.368
Mitochondrial ATP production (OCR, pmol/min)	48.53 (27.69)	44.51 (26.07)	**0.034**
Mitochondrial maximal respiration (OCR, pmol/min)	91.86 (56.41)	86.87 (56.08)	0.226
Mitochondrial spare respiratory capacity (OCR, pmol/min)	34.81 (30.04)	31.71 (28.45)	0.147
**Mitochondrial oxidative stress parameters**			
Log Mitochondrial oxidative stress/mass ratio	− 0.96 (0.21)	− 0.92 (0.24)	**0.049**

Data are presented as mean (standard deviation) and are compared between normal and MCI participants by Student’s t-test. Bold values represent a statistical significance.

*ATP* adenosine triphosphate, *OCR* oxygen consumption rate.

Furthermore, by assessing mitochondrial oxidative stress, we found that log mitochondria oxidative stress/mass ratio was significantly higher in participants with MCI than those with normal cognition.

### Mitochondrial dysfunction in PBMCs was independently associated with cognitive status

As data from the univariate analysis showed an association between cognitive impairment, mitochondrial-linked ATP production, and mitochondrial oxidative stress/mass ratio, we performed a multivariate analysis to determine the association between cognitive impairment vs. other mitochondrial function parameters, including proton leak and mitochondrial respiratory respiratory capacity as shown in Table 3. The unadjusted multivariate analysis in the Table 3 is the multivariate model including five selected mitochondrial function variables before adjusted with the confounding factors. On the other hand, the univariate analysis is the association of each mitochondrial function variables with the MCI. The results showed that reduction of mitochondrial-linked ATP production and elevation of mitochondrial proton leak were associated with MCI (odds ratio = 0.990 (0.981–0.999), p = 0.030 and 1.021 (1.004–1.038), p = 0.016, respectively). Although log mitochondrial oxidative stress/mass ratio did not reach a significant level, the result suggested that an increase in this parameter were associated with cognitive impairment (odds ratio 1.907 (0.961–3.783), p = 0.065). Finally, the factors that may influence cognitive function or mitochondrial function, were adjusted, including age, sex, education level, eGFR, albumin/globulin ratio, WHR, plasma glucose, SBP, DBP, triglyceride, HDL-C, and LDL-C. The results demonstrated that increasing mitochondrial proton leak and log mitochondrial oxidative stress/mass ratio were associated with cognitive impairment (1.027 (1.009–1.046), p = 0.003, and 2.391 (1.119–5.109), p = 0.024, respectively). After adjusting the factors, a reduction in mitochondrial ATP production tended to be associated with MCI (0.990 (0.980–1.000), p = 0.054). Collectively, our results showed that impaired mitochondrial ATP production, increased proton leak in mitochondria, and increased production of oxidative stress by mitochondria were associated with cognitive impairment in participants.

**Table 3:** Binary logistic regression analysis of association between mitochondrial respiration parameters and mitochondrial oxidative stress parameters with odds to become MCI.

Variable	Univariate analysis		Multivariate analysis			
	Odd ratio (95% CI)	p-value	Unadjusted model		Adjusted model	
			Odd ratio (95% CI)	p-value	Odd ratio (95% CI)	p-value
**Mitochondrial respiration parameters**						
Non-mitochondrial respiration	0.999 (0.985–1.013)	0.857	1.008 (0.988–1.029)	0.434	1.004 (0.982–1.027)	0.719
Mitochondrial Basal respiration	0.998 (0.994–1.003)	0.476				
Mitochondrial ATP production	0.994 (0.989–1.000)	0.035	0.990 (0.981–0.999)	0.030	0.990 (0.980–1.000)	**0.054**
Mitochondrial proton leak	1.006 (0.993–1.018)	0.369	1.021 (1.004–1.038)	0.016	1.027 (1.009 -1.046)	**0.003**
Mitochondrial maximal respiration	0.998 (0.996–1.001)	0.227				
Mitochondrial respiratory respiratory capacity	0.996 (0.991–1.001)	0.148	0.997 (0.990–1.003)	0.345	0.996 (0.988–1.003)	0.281
**Mitochondrial oxidative stress parameters**						
Log Mitochondrial oxidative stress/mass ratio	1.808 (1.002–3.261)	0.049	1.907 (0.961–3.783)	0.065	2.391 (1.119–5.109)	**0.024**

Mitochondrial respiration parameters and mitochondrial oxidative stress parameters was used in binary logistic regression model as independent variable. In adjusted model, age, sex, education level, eGFR, albumin/globulin ratio, waist to hip ratio, plasma glucose, systolic blood pressure, diastolic blood pressure, triglyceride, HDL-C, and LDL-C were added into the model as covariate. Bold values represent a statistical significance.

*ATP* adenosine triphosphate, *CI* confidential interval, *HDL-C* high-density lipoprotein cholesterol, *LDL-C* low-density lipoprotein cholesterol.

## Discussion

The major findings of this study are that: (1) cognitive impairment was associated with mitochondrial dysfunction in PBMCs, and (2) by using multivariate binary logistic regression analysis, MCI was associated with lower mitochondria ATP production, more mitochondria proton leak, and higher oxidative stress produced from mitochondria.

The number of aging people is rising continually, and our results showed that age has a strong and independent association with cognitive function. A report from another cross-sectional study demonstrated that elderly subjects (age > 65) with MCI, very mild dementia and dementia have problems with mobility, self-care, usual activities, pain, anxiety, and depression^19^. Thus, the monitoring of cognitive impairment is essential. The MoCA is an assessment tool for MCI, and it is valid for patients with various diseases such as cerebrovascular diseases^20^, multiple sclerosis^21^, and aging^5^. Although MoCA is a screening tool for MCI or dementia, it cannot use for definite diagnosis. However, it is proper as a tool for cognitive assessment in large population (1199 participants) in the present study. In addition, previous studies demonstrated that MoCA has been reliable and valid for screening diagnosis of MCI in Thai population^22,23^. MoCA score has also been the good reliability on MCI screening for dementia, Parkinson’s disease, ischemic cerebrovascular disease, stroke, and transient ischemic attack^24–26^. The prevalence of MCI in the present study was similar with the other previous study, which demonstrated that 71.4% of older Thai people (mean age = 68.3 ± 6.82 years old, n = 482) had MCI^27^. Griffiths and colleagues also suggested that MoCA-Thai version was utilized to screen for MCI in older Thais. Age is a well-established risk factor for cognitive impairment. In this study, the age of the normal cognition group was significantly lower than the MCI group. In addition, education level was also associated with the categorization of the participant by MoCA score. In the normal cognition group, there was a greater proportion of participants with higher education when compared to the MCI group. Furthermore, the number of participants with hypertension was higher in the MCI group. To assess other possible conditions that might affect the cognitive function or mitochondrial function of the participant, general blood parameters were determined. HDL-C and LDL-C levels were significantly lower in the participants with MCI, which may relate to dysregulation of lipid metabolism in the participants with impaired cognitive status. The level of blood glucose was significantly higher in the MCI group, which reflected the impairment in glucose metabolism of the participants, which is highly correlated with cognitive impairment^28^. Several studies revealed that a reduction in albumin/globulin ratio and eGFR were associated with cognitive dysfunction^29–31^. These data indicated that our EGAT participants with MCI were older, and had higher BP and WHR, than those in normal cognition group. These data suggested that metabolic alterations were observed in our MCI participants. Due to these distinct characteristics between the cognitive normal and MCI group, these factors were adjusted in multivariate analysis.

In this study, we focused on mitochondrial function in PBMCs. Mitochondria are the main source of energy or ATP for cells, including neurons. Mitochondria themselves are also the source of ROS. It has been shown that mitochondria in the peripheral cells can indicate early oxidative stress in the progression of Alzheimer’s disease (AD), which is similar to what occurs in the brain^32^. In addition, Sultana and colleagues reported that mitochondria in lymphocytes are peripheral biomarkers in the progression of AD^33,34^. However, only a few studies have investigated the impact of peripheral mitochondrial function in association with MCI in an elderly population. The results showed that MCI is associated with decreasing ATP production and increasing proton leak. In the adjusted model, these associations remain in the same direction, even though ATP production cannot reach a significant level. The basal respiration, maximal respiration, respiratory capacity, and non-mitochondrial respiration were not different between groups. These results suggested that disturbance in ATP production and overproduction of oxidative stress in the peripheral mitochondria would be involved in early pathological changes in the patients with cognitive dysfunction. However, the study from Claus Desler^35^ showed that there was no significant difference between mitochondrial basal respiration, ATP turnover, or spare respiratory capacity in PBMCs. The lower number of participants in that study may be the reason for this inconsistency. According to that study protocol, none of the participants were diagnosed with AD, dementia, or MCI, and the comparison was done between the groups with relative cognitive impairment or improvement in normal cognitive participants^35^.

Besides the mitochondrial respiration, oxidative stress was also significantly higher in the MCI group when compared to the normal cognition group. After adjustment with potential confounding factors, the results were statistically significant. This means that oxidative stress production in mitochondria is independently associated with the cognitive status of participants. Our result was supported by several studies which found an increase in oxidative stress in the lymphocytes of AD patients^36,37^. Our results indicated that even though the patients were in MCI state, the overproduction of oxidative stress by PBMCs’ mitochondria already occur.

We concluded that the mitochondria dysfunction in PBMCs was associated with early cognitive impairment, such as a person with MCI. By multivariate analysis, we showed that mitochondria dysfunction in PBMCs has been independently associated with cognitive impairment. Therefore, the results from this study reveal the association of mitochondrial function in PBMCs and the cognitive impairment in MCI. The causative association or the prognostic prediction among mitochondrial function and cognitive function should be further investigated based on explored variables in this study.

The parameters indicating mitochondria dysfunction in this study show consistency in their association with cognitive function, however, some of the results were nearly statistically significant. The inhomogeneity of participants in this study may lead to the high variability of collected data which needs a greater number of participants to show stronger statistical association. In addition, the causality of the result cannot be established due to the cross-sectional analysis. Future studies with the prospective investigation and more restrictive inclusion criteria for the participants should be performed.

## Materials and methods

### Participants in this study

**Figure 1:**
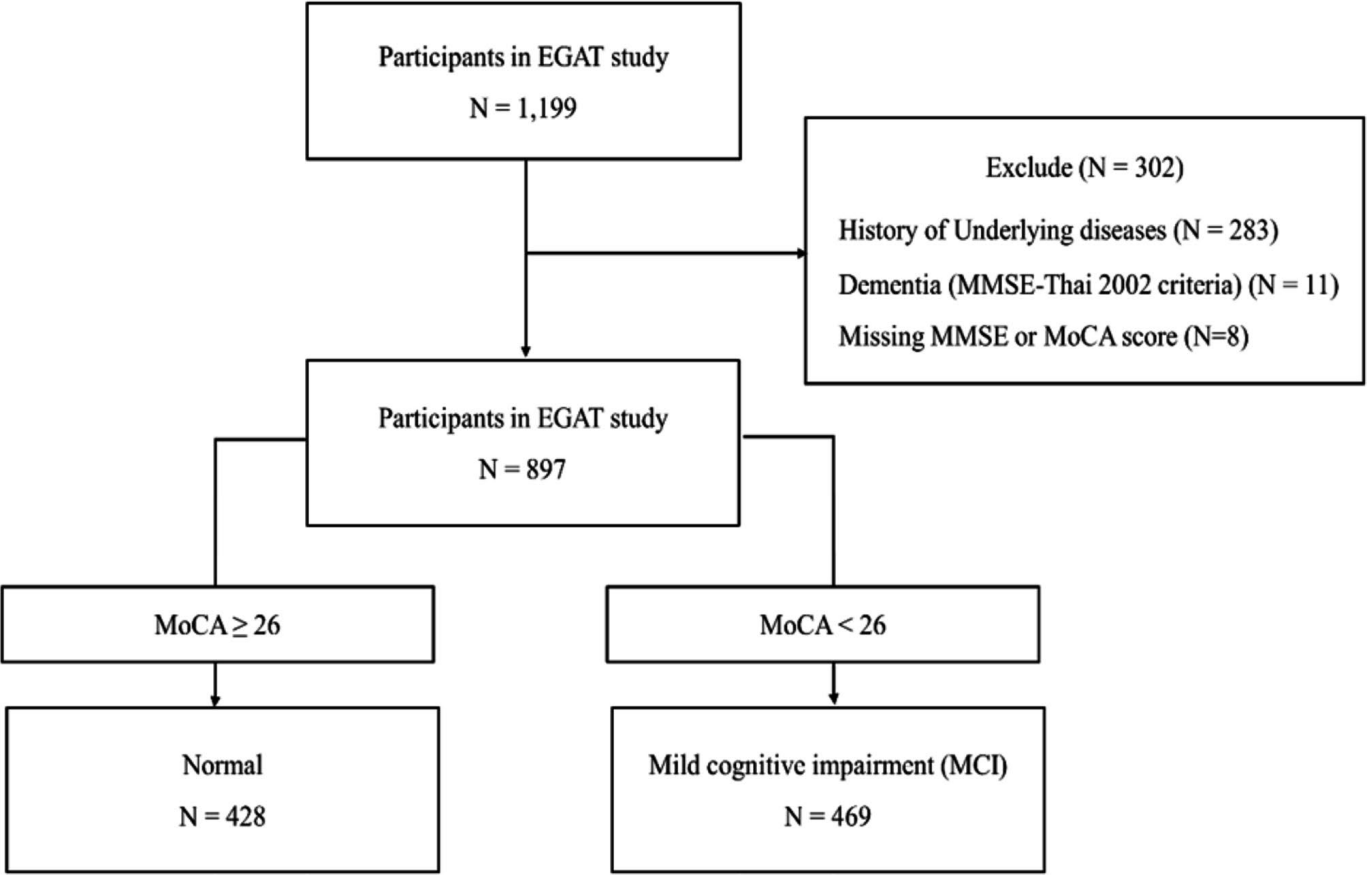
Study protocol for this study. 897 participants from the EGAT cohort were classified to be in the normal cognition group (n = 428) or mild cognitive impairment group (n = 496), according to their MoCA score, and the association between cognitive function and mitochondrial function in PBMCs were determined.

The present study is a cross-sectional study, and it was approved by the Committee on Human Rights Related to Research Involving Human Subjects, Faculty of Medicine Ramathibodi Hospital, Mahidol University, Thailand (Approval number: ID 05-51-19). All experiments involving human participants were carried out under the ethical standards of the institutional and/or national research committee, the 1964 Helsinki Declaration, and its later amendments or comparable ethical standards, with a STROBE statement for reporting data from a cross-sectional study. Participants gave written informed consent. The participants with the age greater or equal to 65 years old who underwent routine physical checkup during August–October 2017 were included to this study. The total participants in the present study were 1199 subjects. The exclusion criteria were (1) history of following underlying diseases; stroke (ischemic or hemorrhagic), cardiovascular diseases (coronary artery disease, myocardial infarction, heart failure, peripheral arterial disease), Parkinson’s disease, kidney disease, liver disease, systemic lupus erythematosus, rheumatoid arthritis, thyroid diseases, cancer, (2) Dementia (according to MMSE-Thai 2002 criteria), (3) Missing MoCA score data. The MoCA assessment was used to determine the cognitive function in all subjects. There were no illiterate participants in this study, and they received at least the primary education. Activities of daily living (ADL) and instrumental activities of daily living (IADL) were assessed in all participants. SBP and DBP were also recorded. BMI and WHR were measured. Diabetes mellitus, hypertension, and dyslipidemia status were asked. Then, the blood was collected, and the blood chemistry was analyzed, including blood glucose, HDL-C, LDL-C, VLDL-C, aspartate aminotransferase (AST), alanine transaminase (ALT), alkaline phosphatase (ALP), and eGFR. The glomerular filtration rate (GFR) in this study was estimated by Chronic Kidney Disease Epidemiology Collaboration (CKD-EPI) equation. eGFR = 141 × min(S_cr_/κ, 1)^α^ × max(S_cr_/κ, 1)^−1.209^ × 0.993^Age^ × 1.018 (if female) × 1.159 (if black) where the S_cr_ stands for serum creatinine (mg/dL), the κ stands for 0.7 for females and 0.9 for males, and the α stands for − 0.329 for females and − 0.411 for males. Six mL samples of blood were kept in Ethylenediaminetetraacetic acid (EDTA) coated tubes. The PBMCs were isolated from the EDTA-blood. PBMCs were stained with fluorescent dyes to determine mitochondrial oxidative stress and mass, and they were analyzed by flow cytometry. Mitochondrial respiration was measured using an extracellular flux analyzer, and mitochondrial-linked ATP production was analyzed automatically. The flow chart of the study protocol is shown in Fig. 1.

### MoCA

MoCA is an assessment tool for screening MCI in the elderly. The MoCA assesses eight types of cognitive ability including orientation, short-term memory/delay recall, executive function/visuospatial ability, language ability, abstraction, animal naming, and a clock-drawing test. The MoCA scores range from 0 to 30, and a score equal to or higher than 26 is usually considered normal cognition^6^. Participants receiving a score below 26 were in the MCI group. MoCA testing was performed by specialist nurses and validated by Board-certified Geriatricians. The educational levels have been adjusted in MoCA scores of the present study.

### PBMCs isolation

The EDTA-blood was centrifuged, and the plasma was removed. Then, the blood was overlayered on the Ficoll (Histopaque, Sigma-Aldrich, Missouri, USA), the ratio of blood to the Ficoll was 2:1. The layer was centrifuged at 400*g* for 30 min, and the PBMCs were collected at the plasma-Ficoll interface^38^.

### Mitochondrial oxidative stress and mass in PBMCs

2 × 10^5^ PBMCs in Hanks’ buffer salt solution were stained with 5 μM of MitoSOX dye (Invitrogen, California, USA), and co-incubated with 100 nM of MitoTracker Green dye (Invitrogen, California, USA). PBMCs were incubated with the dyes at 37 °C for 30 min. Then, the cells were washed and subjected to flow cytometry analysis (BD FACS Celesta, BD biosciences, California, USA). The mean fluorescent intensity of MitoSOX and MitoTracker Green dyes were automatically analyzed by BD FACS Diva version 6. (BD biosciences, California, USA)^39^. The ratio of mean fluorescent intensity of MitoSOX/MitoTracker was manually calculated^39^.

### Mitochondrial respiration in PBMCs

2 × 10^5^ PBMCs were loaded onto 96 XFe cell plates (Seahorse, Agilent, California, USA), and supplemented with an XF base medium containing 1 mM pyruvate, 2 mM glutamine, and 10 mM glucose. Mitochondrial respiration was determined using a seahorse XFe 96 analyzer (Seahorse, Agilent, California, USA). First, the basal respiration was determined, then 1 µM of oligomycin, 2 µM of Trifluoromethoxy carbonylcyanide phenylhydrazone (FCCP), and 0.5 µM of mixed rotenone and antimycin were added, sequentially. Mitochondrial respiration parameters were automatically analyzed using the Wave version 2.6 program (Agilent, California, USA)^40^.

### Statistical analysis

Statistical analysis was done by IBM SPSS software version 23.0 (IBM Corp., Armonk, New York, USA). Continuous variables were expressed as the mean with standard deviation. Comparisons between groups were performed by independent sample Student’s t-test or Chi’s square test for continuous variables and categorical variables, respectively. The participants were classified according to their MoCA score to have normal cognition or mild cognitive impairment. The association of mitochondrial respiration parameters and mitochondrial oxidative stress parameters with the cognitive status of the participant was assessed by binary logistic regression analysis. Age, sex, education level, eGFR, albumin/globulin ratio, waist to hip ratio, plasma glucose, systolic blood pressure, diastolic blood pressure, triglyceride, HDL-C, and LDL-C were included as covariates in the adjusted model. Mitochondrial oxidative stress/mass ratio was Log transformed before included in the model due to the non-normal distribution of data. Odds ratios and 95% confidential interval of risk to become MCI by each parameter were noted. A p-value of less than 0.05 was considered statistically significant.

## Data Availability

The datasets generated during this study are available from the corresponding author on a reasonable request.
